# HARMONIZATION OF CHRONIC CONDITIONS WAREHOUSE DATA FOR OUTCOMES RESEARCH ON GERIATRIC SYNDROMES

**DOI:** 10.1093/geroni/igad104.1465

**Published:** 2023-12-21

**Authors:** Suzan Ahmad, Hyosin Kim, Soko Setoguchi, Fred Kobylarz, Anum Zafar, Maria Lopez, Haiqun Lin, Olga Jarrín

**Affiliations:** Rutgers, The State University of New Jersey, Newark, New Jersey, United States; Oregon State University, Corvallis, Oregon, United States; Rutgers, The State University of New Jersey, New Brunswick, New Jersey, United States; Rutgers, The State University of New Jersey, New Brunswick, New Jersey, United States; Rutgers, The State University of New Jersey, New Brunswick, New Jersey, United States; Rutgers, The State University of New Jersey, New Brunswick, New Jersey, United States; Rutgers, The State University of New Jersey, Newark, New Jersey, United States; Rutgers, The State University of New Jersey, New Brunswick, New Jersey, United States

## Abstract

Geriatric syndromes such as cognitive impairment, falls, depression, urinary incontinence, pressure ulcers, and polypharmacy are common conditions and syndromes that affect older adults. Studying geriatric syndromes in Medicare beneficiaries may provide important insights for improving health outcomes and quality of life. As a result of the Improving Medicare Post-Acute Care Transformation Act of 2014, it is now easier to compare standardized geriatric assessment data (including information about geriatric syndromes) collected in post-acute and long-term care settings with corresponding data from claims, encounter, Part D, and durable medical equipment files. However, to our knowledge, methods to link and harmonize post-acute and long-term care assessment data (available for 75% of decedents) are yet to be utilized in the health services research community. The goal of this project is to develop harmonized datasets that can be used to identify geriatric syndromes and study their prevalence, cost, health outcomes, and service delivery factors over time. This work will allow our team and other researchers to fully leverage these data for use in predictive algorithms and artificial intelligence and machine learning (AI/ML) research. The project uses the five most recent years of data available from the Centers for Medicare and Medicaid Services (CMS) Chronic Conditions Data Warehouse. Data sources include Medicare and Medicaid claims, encounter, MedPAR, Hospice claims, Minimum Data Set (MDS), MDS-Swing Bed (SB), Outcome and Assessment Information Set (OASIS), Inpatient Rehabilitation Facility Patient Assessment Instrument (IRF-PAI), Pharmacy (Part D), and Durable Medical Equipment (DME).

